# Within the Wards

**Published:** 1888-08-04

**Authors:** 


### WITHIN THE WARDS.

### DANGEROUS ABUSE OF A NEW DRUG.

THE *Brooklyn Medical Journal* (U.S.A.) states that antipyrin is being very generally taken without the advice of a qualified medical practitioner. Both physicians and druggists report upon the alarming frequency of its use. It is said to be a not uncommon thing for those who suffer from headaches to purchase the drug, and take it in twenty-grain doses, entirely unconscious that they run any risk in so doing. Evidence is accumulating that the use of antipyrin in these circumstances is fraught with danger. There are already a considerable number of cases recorded of alarming symptoms having been produced by even small doses, so that physicians themselves never use the drug without due caution. It cannot be too often repeated that self-medication with the dangerous drugs now so frequently prescribed by medical men is an eminently dangerous practice.

### TWO BULLETS IN A PATIENT'S BRAIN.

The following case is recorded by Dr. George R. Fowler, of Brooklyn. A man, aged thirty-five, entered St. Mary's Hospital on May 13th, 1887. A pistol had been fired at him, the muzzle almost touching his forehead. Two bullets entered the brain, one passing through the frontal bone to the left of the median line, the other entering at the inner canthus of the right eye, tearing away the lower and posterior portion of the frontal sinus of that side. In order, if possible, to discover and extract the bullets, a crucial incision was made and the bone laid bare. A compound fracture of the frontal bone was revealed. One bullet had passed downwards, perforating the dura mater, and was lost in the cerebrum. It had left an opening in the dura large enough to admit a portion of the little finger. The second bullet was found difficult to trace. It had smashed and destroyed the posterior wall of the right frontal sinus, carrying portions of it into the cerebrum, from whence they were removed. A portion of the brain matter escaped; that part of the frontal lobe which rested on the roof of the orbit on the right side was considerably lacerated, and some of it destroyed. An attempt was made to locate the balls by drawing an imaginary line across in a line with the pistol and trephining posteriorly where that line would meet. Through the opening made in the occipital bone a small needle was systematically passed in radiating directions into the brain substance. By this means the brain was explored in almost every part, but without success—neither of the bullets being found. The wound was then dressed. In front, where the bullets had entered, the débris was first removed, and then about a dozen strands of catgut were passed into the wound, coming through the soft tissues over the destroyed part of the frontal bone. Half-a-dozen strands were also passed through what remained of the frontal sinus to afford an escape at that point. The left nasal cavity was irrigated with solution of mercuric iodide, cleansed, and stuffed with iodoform cotton, and the dressing completed antiseptically. The strangest part of the story remains to be told. The patient entirely recovered without a single troublesome symptom; he never even complained of a headache. He was up and about, and helping with the work of the ward in three weeks. He left the hospital on June 7th (having entered it less than four weeks before) entirely healed, and in full possession of all his faculties. He returned once or twice with small portions of dead bone requiring removal, but is now quite well, suffering nothing apparently from his injuries beyond a slight occasional depression of spirits. Both bullets are still somewhere in the brain. The case is recorded to show how tolerant certain portions of the brain tissue are of injuries, and of the permanent presence of foreign bodies.

### SUCCESSFUL EXTIRPATION OF A CANCEROUS LARYNX.

At the present time, when the late Emperor Frederick's case is still fresh in the memory, it is opportune to record a case of successful operation for the removal of the larynx. The case was published in the *British Medical Journal* of July 21st, 1888. The patient was a man aged 62, a native of Germany, residing at St. Kilda, Victoria. He began to lose his voice in the early part of 1887, and consulted Dr. Cox, of Melbourne. Dr. Cox discovered a smooth growth below the left vocal cord. The patient, however, refused to be treated for it at that time. Not long afterwards the voice returned, and the power of speech continued for about four months. Then the hoarseness reappeared, and was accompanied by cough, spasmodic seizures, and inability to lie down. On October 1st a laryngoscopic examination was made, and it was discovered that there was a small ulcer below the left vocal cord. Dr. W. Gardner, of Adelaide, saw the patient with Dr. Cox, and the two medical men came to the conclusion that the disease was cancer of the larynx. Dr. Cox then removed a small portion of the growth with the laryngeal forceps, which he submitted for microscopic scrutiny to Professor Allen, who pronounced it to be epitheliomatous cancer. The patient now consented to an operation; and on the following day the whole of the larynx was removed by Dr. Gardner, assisted by Drs. Cox and Yorke. The day after the operation the tracheotomy tube became accidentally blocked, and the patient all but died of suffocation. But Dr. Yorke sucked the tube clear and performed artificial respiration with such energy and promptitude that life was restored. After this alarming accident the progress of recovery was uninterrupted. On October 10th, nine days after the operation, jelly was taken by the mouth, and on the day following the patient was able to get up. On Christmas Day, less than three months from the date of extirpation, he walked into the city without attendance. The patient was present and his case examined by the members of the Victoria Branch of the British Medical Association on April 25th of the present year. He was in excellent health, and could speak in whispers sufficiently distinct to make himself understood. There was then no indication whatever of any return of the growth.

